# Linking heart rate variability to psychological health and brain structure in adolescents with and without conduct disorder

**DOI:** 10.3389/fpsyt.2023.1101064

**Published:** 2023-06-27

**Authors:** Ana Cubillo, Antonia Tkalcec, Helena Oldenhof, Eva Unternaehrer, Nora Raschle, Gregor Kohls, Lucres Nauta-Jansen, Amaia Hervas, Aranzazu Fernandez-Rivas, Kerstin Konrad, Arne Popma, Christine Freitag, Stephane de Brito, Graeme Fairchild, Christina Stadler

**Affiliations:** ^1^Department of Child and Adolescent Psychiatry (research section), University Psychiatric Clinics, Basel, Switzerland; ^2^Zurich Center for Neuroeconomics, Department of Economics, University of Zurich, Zurich, Switzerland; ^3^Amsterdam UMC, Vrije Universiteit Amsterdam, Department of Child and Adolescent Psychiatry, Amsterdam Public Health, Amsterdam, Netherlands; ^4^Jacobs Center for Productive Youth Development, University of Zurich, Zurich, Switzerland; ^5^Department of Child and Adolescent Psychiatry, Faculty of Medicine, TU Dresden, Dresden, Germany; ^6^Hospital Universitario Mutua Terrassa, IGAIN, Barcelona, Spain; ^7^Biocruces Bizkaia Health Research Institute, Basurto University Hospital, University of the Basque Country, Bilbao, Spain; ^8^RWTH Aachen University & JARA-Brain Institute, Aachen, Germany; ^9^Child and Adolescent Psychiatry Amsterdam University Medical Centers, Amsterdam, Netherlands; ^10^Child and Adolescent Psychiatry, Goethe University, Frankfurt, Germany; ^11^University of Birmingham, Birmingham, United Kingdom; ^12^Developmental Psychopathology, University of Bath, Bath, United Kingdom

**Keywords:** heart rate variabiity, emotion regualtion, self-regulation, brain structure, central autonomic network, conduct disorder

## Abstract

**Aims:**

Heart rate variability (HRV) measures have been suggested in healthy individuals as a potential index of self-regulation skills, which include both cognitive and emotion regulation aspects. Studies in patients with a range of psychiatric disorders have however mostly focused on the potential association between abnormally low HRV at rest and specifically emotion regulation difficulties. Emotion regulation deficits have been reported in patients with Conduct Disorder (CD) however, the association between these emotion regulation deficits and HRV measures has yet to be fully understood. This study investigates (i) the specificity of the association between HRV and emotion regulation skills in adolescents with and without CD and (ii) the association between HRV and grey matter brain volumes in key areas of the central autonomic network which are involved in self-regulation processes, such as insula, lateral/medial prefrontal cortices or amygdala.

**Methods:**

Respiratory sinus arrhythmia (RSA) measures of HRV were collected from adolescents aged between 9–18 years (693 CD (427F)/753 typically developing youth (TD) (500F)), as part of a European multi-site project (FemNAT-CD). The Inverse Efficiency Score, a speed-accuracy trade-off measure, was calculated to assess emotion and cognitive regulation abilities during an Emotional Go/NoGo task. The association between RSA and task performance was tested using multilevel regression models. T1-weighted structural MRI data were included for a subset of 577 participants (257 CD (125F); 320 TD (186F)). The CerebroMatic toolbox was used to create customised Tissue Probability Maps and DARTEL templates, and CAT12 to segment brain images, followed by a 2 × 2 (sex × group) full factorial ANOVA with RSA as regressor of interest.

**Results:**

There were no significant associations between RSA and task performance, neither during emotion regulation nor during cognitive regulation trials. RSA was however positively correlated with regional grey matter volume in the left insula (pFWE = 0.011) across all subjects.

**Conclusion:**

RSA was related to increased grey matter volume in the left insula across all subjects. Our results thus suggest that low RSA at rest might be a contributing or predisposing factor for potential self-regulation difficulties. Given the insula’s role in both emotional and cognitive regulation processes, these brain structural differences might impact either of those.

## 1. Introduction

Heart Rate Variability (HRV) is a psychophysiological measure that captures beat-to beat vagal modulation of the heart rate. It has a relatively stable, trait-like character and has consistently been associated with physical and psychological wellbeing (1–7). Autonomic system adjustment to contextual demands is regulated by the Central Autonomic Network (CAN), a brain network which receives input from both the sympathetic and parasympathetic branches of the Autonomic Nervous System (ANS) underlying not only the control of autonomic responses but also visceromotor, neuroendocrine, and behavioural responses (7–12). Key components of the CAN include the anterior cingulate, insula, orbitofrontal and ventromedial prefrontal cortices, together with amygdala, thalamus, subthalamic, and brain stem nuclei (9, 11, 12). These structures are not only involved in autonomic regulation but also in the implementation of emotional and cognitive self-regulation processes (10, 13–17). Furthermore, the Neurovisceral Integration Model postulates HRV as an index of functional integrity of brain structures involved in higher executive functions including working memory, inhibitory control as well as in emotion regulation (10, 15). Measures of vagal function such as HRV, primarily mediated by parasympathetic vagal innervation, can also index inhibitory prefrontal processes involved in stress response, being therefore indicative of the ability of the autonomic system to respond and flexibly adjust to contextual demands (7, 8).

Individuals with low HRV show heightened reactivity to emotional stimuli (1) as well as difficulties in emotion regulation and impulse control in daily life (18). In line with this, recent evidence from meta-analytic studies has shown that low HRV measures are observed across several different psychiatric disorders (4, 19–21), including Conduct Disorder (CD) (22), hence suggestive of its potential transdiagnostic character. Low HRV has been associated with antisocial and aggressive behaviours (23), callous-unemotional traits (24), depressive symptoms or suicidal ideation (25–28) or anxiety disorders (29), thus supporting the idea of HRV as a potential index of deficits in emotion regulation processes and psychological well-being (7). ANS indicators have indeed long been discussed as potential biomarkers for CD (30), a highly impairing psychiatric disorder emerging in childhood or adolescence characterized by severe antisocial and aggressive behaviour. However, evidence from a recent review and meta-analyses (22, 31) have suggested a high physiological heterogeneity, where the association between ANS function and CD or antisocial behaviours might vary as a function of the clinical subtype, as well as of the ANS measures and analytical methods used. How ANS function might be associated with the phenotypic presentation of CD is thus not yet fully understood.

At the neural level, individual differences in HRV measures at rest have shown a positive association with cortical thickness in the dorsal anterior cingulate gyrus in healthy young adults (32) as well as in war veterans (33). However, in healthy adults also negative associations were found between HRV and grey matter volumes in subcortical and limbic regions including the putamen, caudate, amygdala, insula or superior temporal gyrus (34) while others found no association (35). HRV measures have been shown to be highly sensitive, influenced by a number of factors such as sex, age, SES or variations in the assessment methods used (36). Thus, the mechanism underlying the association between brain structures and HRV measures in healthy populations remains unclear.

Patients with a diagnosis of CD have been reported to have abnormal structure and function in regions including the insula, amygdala, temporal cortex and ventral striatum (30, 37–41). This association has been frequently discussed in the context of emotion regulation deficits (40, 42–45). However, children and youths diagnosed with CD show highly heterogeneous symptomatic (30), neurocognitive (46), and physiological profiles (22). The Research Domain Criteria (RDoC) (47) adopts a dimensional approach in which specific alterations in predefined biological systems lead to various symptomatic presentation. The RDoC approach could therefore contribute to improve the understanding of the high heterogeneity present in neuropsychiatric disorders, as in CD. Thus, psychophysiological measures have been suggested as having the potential to help disentangle the heterogeneity within CD populations. Given the association between HRV and emotion regulation deficits, HRV has been discussed as a potential physiological marker for CD. However, recent studies using different HRV indices such as Respiratory Sinus Arrythmia (RSA) or Pre-ejection Period (PeP) did not find significant associations between ANS activity and CD (48) or antisocial behaviours considered from a dimensional perspective (49). While different psychophysiological measures can be closely interrelated, subtle differences between groups with small effect sizes might be overlooked depending on the measure selected. Furthermore, the association between HRV and emotion regulation in patients with CD might differ significantly based on symptomatic presentations or comorbidities (31, 50). Thus, altered HRV has been shown in patients with CD and comorbid internalizing disorders, but not in those with callous-unemotional traits (31). In addition, different factors such as Body-Mass Index (BMI), socio-economic status (SES), medication intake, sports (48, 49), the type of task and analyses used or the physiological outcome investigated (22) might influence HRV measures hence contributing to the heterogeneous body of evidence.

Most of the research studies on the role of HRV in patients with CD have focused on its potential association with emotion regulation deficits (31). However, HRV might be associated with more generic self-regulation skills (51, 52). Successful self-regulation skills are crucial for goal-directed behaviour and require the implementation of selective and sustained attention, cognitive control and inhibition of inadequate emotional and behavioural responses (53). Consequently, successful self-regulation skills require the ability to regulate both emotional and cognitive processes. The available evidence seems to suggest that HRV could indeed be better understood as a general index of regulatory processes, as it has been shown to be associated with top-down self-regulation abilities (51), executive function (54), and performance during inhibition or switch tasks (52). However, the effect might be rather small and moderated by a number of variables, some inherent to the individuals such as age or sex, others related to the metrics used or the methodological approaches followed during the assessments (52).

In summary, the evidence on the association between HRV and emotion regulation capacities in the few existing studies in patients with CD is not consistent (22). Thus, the present study investigates whether HRV might be associated with more generic self-regulation deficits and with individual differences in key components of the CAN, and whether these associations differ between healthy youths and youths with CD. To test this, we capitalized on data collected as part of a European multicenter study (FemNAT-CD Project), which combines psychophysiological (with HRV operationalized via RSA), neuropsychological (performance of an Emotional Go/NoGo task) and brain structure data in a group of male and female adolescents with CD, as well as of typically developing adolescents. This allowed us to investigate whether there are group differences between adolescents with CD and healthy peers in a) the association between RSA and emotion regulation and cognitive regulation performance during an Emotional Go/NoGo task and b) the potential association between the HRV measures and grey matter volumes in those regions typically defined as part of the CAN (9). Based on the available evidence on factors that might potentially influence the association between CD and HRV, we took those variables into consideration including comorbid internalising symptoms or CU traits within the CD group (31), as well as age, sex, SES, BMI, cigarette consumption, involvement in sport or IQ (48, 55). We hypothesized that if RSA can be considered as a potential general index of self-regulation, we will find that: (1) there will be a positive association between task performance in both the emotional and cognitive regulation conditions of the Emotional Go/NoGo task and RSA measures across all participants, without significant group differences between patients and controls, and (2) RSA measures will be positively associated with cortical grey matter volumes in brain regions that are part of the CAN network across all participants, without significant group differences between patients and controls.

## 2. Materials and methods

### 2.1. Participants

Participants of this study were part of the *Neurobiology and Treatment of Adolescent Female Conduct Disorder* (FemNAT-CD) project^1^, a large European multicenter study investigating gender differences in the neurobiology underlying CD. The final sample (*N* = 1,446) of this study included 693 CD (427 females) and 753 TD (500 females) youth aged 9 to 18 years (M = 14.36; SD = 2.44 years) who had valid psychophysiological and neuropsychological data. In addition, structural MRI data was available for a subset of participants (*N* = 577, CD: *n* = 257, 125 females; TD: *n* = 320, 186 females). Further details regarding the sample characteristics are reported in Table 1.

**Table 1:** Demographics and clinical characteristics of the participants.

	TD (*N* = 753)		CD (*N* = 693)		*t*-value	*p*-value
	Mean (SD)/sum	NR missing values	Mean (SD)/sum	NR missing values		
Sex	500F/253M	0	427F/266M	0	−1.755	0.080
Age (Years)	14.138 (2.477)	0	14.385 (2.285)	0	−3.451	0.001
IQ score	103.695 (12.363)	12	94.665 (12.465)	46	−5.134	<0.001
CU traits	16.809 (7.594)	0	33.78 (11.867)	28	21.424	<0.001
Internalising symptoms	5.772 (5.814)	125	13.68 (9.912)	212	8.742	<0.001
ADHD	0%	4	31.9%	6	333.2	<0.001
PNS medication intake	3.2%	5	32.6%	8	8.542	<0.001
BMI	20.743 (4.179)	72	22.158 (4.578)	93	1.737	0.083
SES	0.345 (0.91)	28	−0.412 (0.949)	88	−6.133	<0.001
Cigarettes per day	0 (2)	49	5 (7)	53	7.872	<0.001
Sports (h)/week	4.663 (4.637)	121	3.72 (4.357)	116	−2.656	0.008

The table shows the mean and standard deviation for each group as well as the group differences for the key demographic variables and symptomatic scores utilized in the present study. For each variable and within each group, the number of participants with missing value for the corresponding value is shown. Missing values were imputed using the MICE package in R with 5,000 imputations using all of the available data for those participants used in the present study. Between group comparisons in the key variables shown in the table were conducted after imputation, and they do not differ from those before the imputation. CD, conduct disorder group; TD, typically developing adolescents; CU traits, Callous Unemotional Traits total score of the ICU questionnaire; Internalising symptoms, total score on internalising symptoms subscale of the parent reported CBCL questionnaire; ADHD, ADHD criteria fulfilment according to the DSM-5 coded as a binary variable with 1/0 indicating the fulfillment/not fulfillment of criteria for a diagnosis of ADHD at the time of the interview; IQ, intelligence quotient (total score of the WASI, WISC or WAIS); PNS medication intake, indicates the number of individuals with a positive intake of medication affecting Parasympathetic Nervous System (PNS) activity at the time of the assessment; BMI, body-mass-index; SES, socio- economic status based on parental income, education and occupation. Significant group difference = *p* < 0.05. Except for sex and BMI, all other variables significantly differed between groups.

Participants were recruited using flyers and advertisement in clinics, youth welfare centers, internet forums and schools, in Birmingham and Southampton (UK), Bilbao and Barcelona (Spain), Amsterdam (Netherlands), Aachen and Frankfurt (Germany), Szeged (Hungary), Athens (Greece), and Basel (Switzerland) between 2003 and 2013. CD youth had to fulfil DSM-5 diagnostic criteria for CD (56), while TD youth were included if they did not meet criteria for any current psychiatric disorder and had no previous DSM-IV diagnosis of CD, ODD or ADHD. Additional exclusion criteria for both groups were a diagnosis of autism spectrum disorder or schizophrenia (ICD-10, DSM-IV-TR or DSM-5), current bipolar disorder or mania, monogenetic disorder, genetic syndrome, any chronic or acute neurological disorder, treatment for epilepsy, history of traumatic brain injury, or IQ <70. The FemNAT-CD project was conducted in accordance with the Declaration of Helsinki and approved by the European Commission and local ethics committees of all participating sites. All participants and their caregivers provided written informed assent/consent.

### 2.2. Clinical and behavioural measures

#### 2.2.1. Clinical interviews and questionnaires

Participants and their parents/caregivers were assessed via a semi-structured interview (Schedule for Affective Disorders and Schizophrenia for School-Age Children-Present and Lifetime version, K-SADS-PL) (57). In addition, participants and parents/caregivers completed different questionnaires and behavioural measures (for more information please see (46)).

Internalising symptoms were assessed through the parent report version Child Behavior Checklist (CBCL) (58) and CU traits through the total score from the parent report version of the Inventory of Callous-Unemotional Traits (ICU) (59). Presence of ADHD was assessed during the interview and coded as a binary variable with 0 and 1 indicating the absence/presence of a current diagnosis of ADHD. We additionally accounted for factors with known impact on HRV measures. These included SES, BMI, number of cigarettes smoked per day and physical activity (hours/week) habits, and intake of medication that might affect parasympathetic nervous system (PNS) function (see Table 1). SES scores were computed using principal component extraction based on parental income, education and occupation (ISCO-08) (60) (ISCED) (61), and standardized within each country to avoid potential economic variation at the country level. Current medication intake of compounds that might affect PNS function was assessed by asking the participant, caretaker, therapist, or parent, and coded as a dichotomous variable (yes/no). Depending on age and language, IQ was assessed using the Wechsler Abbreviated Scale of Intelligence (WASI-II) (62), the Wechsler Intelligence Scale for Children (WISC) (63) or the Wechsler Adult Intelligence Scale (WAIS) (64) (further details on the assessments and scoring procedures can be found in (46, 48). Participants performed the emotional Go/NoGo task and underwent a psychophysiological assessment in separate study sessions.

#### 2.2.2. Emotional Go/NoGo task

Performance in the Emotional Go/NoGo task was used to investigate the potential specificity of the association between RSA and self-regulation within the emotional or cognitive domains. This is an adapted version of the Emotional Go/NoGo task developed by Hare and colleagues (65, 66), where on each trial participants are presented with human faces, which might depict a neutral, happy or fearful expression. Trials are presented in blocks of 48, with only two types of emotions presented on each block. Participants are instructed to press a button as fast as possible when they see one of these expressions (Go trials, 73% trials in each block) and refrain from responding when presented with the second emotional expression of the block (NoGo trials, 27% trials in each block). Stimuli duration was 0.5 s, with a fixed 1 s interstimulus interval (for further details, (see 46)). The different combinations of Go/NoGo trials allows the classification of the blocks as indexing emotion regulation (emotional faces as NoGo stimuli in the context of neutral faces as Go-stimuli) or cognitive regulation (neutral faces as NoGo stimuli in the context of emotional faces as Go-stimuli) (66). The main performance measures in the task were response times to Go trials and proportion of correct response to NoGo trials. These were first z-transformed and then combined in a single measure to account for the speed-accuracy trade-off that is commonly observed in the task, with slower response in the Go trials being accompanied typically with a higher proportion of successful NoGo trials (66). Thus, the Inverse Efficiency Scores (IES) (67) were calculated indicating the ratio of z-transformed mean reaction time (Go trials) to z-transformed correct responses to NoGo stimuli (1 – incorrect responses to NoGo trials) (66).

#### 2.2.3. Psychophysiological assessment

The procedures followed to acquire and process the electrocardiogram and respiratory rate (RR) data have been described in detail elsewhere (48). In short, to ensure familiarization with the setting and minimize potential effects of stress, application of H98SG ECG Micropore electrodes was followed by a 10 min habituation period. Next, a 5 min excerpt from an aquatic video (Coral Sea Dreaming, Small World Music Inc.) was presented on a DELL Latitude E5550 Laptop with Sennheiser HD 201 earphones, to obtain a baseline measure for HRV. Prior to the assessment, participants were asked to refrain from smoking (1 h), and from consuming alcohol or drugs (24 h) (A detailed description of the psychophysiological measurement procedure can be found in (48, 49)).

Heart and respiration rate (RR) were assessed to compute RSA. RSA is a common measure for heart-rate variability (HRV) as it indexes parasympathetic activity, which a growing body of research suggests to be linked with emotion regulation capacities (68). Respiration cycle and ECG were recorded using a VU-AMS device (Vrije Universiteit Ambulatory Monitoring System) (69). Raw data was pre-processed using automated and manual steps provided by the VU-DAMS software package version 3.9 to ensure high data quality. RSA was subject to natural-log transformation prior to the analyses to approach a normal distribution of the data (70).

### 2.3. MRI acquisition

Each site followed a site qualification procedure before starting data collection to ensure comparability of MRI data acquisition. Images were acquired using either a Siemens Trio (Frankfurt and Southampton), Siemens Prisma (Aachen and Basel) or Philips scanner (Birmingham) – all at 3 Tesla. Structural T1-weighted magnetization prepared rapid gradient echo (MPRAGE) images were acquired for each participant which included 192 slices, field of view 256 mm, voxel size 1 × 1 × 1 mm, repetition time 1900 ms, echo time 2.42 (Aachen and Basel), 2.74 (Frankfurt), 3.7 (Birmingham), or 4.1 ms (Southampton), flip angle 9 degrees.

### 2.4. Statistical analysis

#### 2.4.1. Behavioural analysis

All analyses were conducted using R (Version 4.1.2) (71) implemented on RStudio (Version 1.4.1717). Missing values on the relevant variables to be included in the models were imputed using the “mice” packed to implement Multiple Imputation by Chained Equations (72). Details on the missing data for each variable are shown in Table 1. All behavioural and clinical variables were z-transformed before being entered in the analyses.

We examined the association between RSA and performance measures in the Emotional Go/NoGo task (IES for emotion regulation and cognitive regulation conditions), with RSA values entered as predictors and task as dependent variables with the main regressors of interest being RSA and group (CD vs. TD).

Multilevel mixed models (MLM) were used for all behavioural data analyses, with the data nested by data collection site to account for dependency in observations using the package nlme (73) and with maximised log-likelihood (‘ML’) as estimation method. Patients with CD often present with comorbid internalising symptoms and CU traits, which have been previously shown to drive psychophysiological heterogeneity (31). To further investigate whether including these variables would improve the data fit, we conducted a model comparison between models without (simple) and with (extended) these regressors. Thus, in the simple model we included the following regressors as variables of no interest: age, sex, comorbid ADHD, SES, number of cigarettes smoked per day and IQ and in the extended model we added two additional regressors to account for the presence of internalising symptoms and CU traits to compare their relative fit to the data. The two models were then compared using the anova.lme command from the nlme package (73). These analyses allowed us to identify whether there was additional variability in the data explained when adding the internalising symptoms and CU traits in the model.

To test the robustness of the results, we repeated the analyses excluding participants whose prescribed medication might have affected their PNS function and therefore, HRV measures.

#### 2.4.2. MRI data preprocessing and analyses

Structural MRI data was analysed using the CAT12 toolbox (74) implemented in SPM12 (75) using MATLAB (v2020b). Data was preprocessed using standard CAT12 steps, and only those individuals whose data quality was classified by CAT12 as C or higher were included in the analyses. Customised tissue probability maps (TPM) across all individuals were created using the CerebroMatic toolbox (76) and used to segment individual data into the different tissues (grey/white matter and CSF) and smoothed using a 8 mm Gaussian kernel. Total Intracranial Volumes (TIV) were then calculated for each participant. The smoothed grey matter volumes were included in a 2 × 2 full factorial ANOVA, with gender and group as factors. Standardized RSA values were included as the main regressor of interest, whereas TIV, Age, IQ, and site (one regressor per site, using one-hot encoding) are used as regressors of no interest. Analyses were masked for the cortical and subcortical regions which are involved in both emotional and cognitive self-regulation, as well as those typically included as part of the CAN (9). These regions included the amygdala, insular cortex, anterior cingulate cortex and medial prefrontal cortex (Supplementary Figure S1). Results are deemed as significant at a *p* < 0.05, family-wise error (FWE) correction, using the Threshold-Free Cluster Enhancement technique (TFCE) with 5,000 permutations.

## 3. Results

### 3.1. Behavioural results

Univariate ANOVA comparisons showed that the group of patients with CD differed from the control participants in several demographic variables including age, IQ, SES, medication intake, and number of cigarettes per day or hours of sports per week, as well as in clinical variables including comorbid ADHD, CU traits and internalising symptoms (Table 1). The results of the initial multilevel regression analyses on RSA showed significant main effects of age, cigarette, and medication use (Supplementary Table S1) which we then included in our main analysis as covariates. When Group was included as a regressor in the model (as a factor with two levels: TD and CD), no significant effect of group was observed, suggestive of no RSA differences between the two groups when potential influencing factors (age, medication intake or cigarette consumption) are taken into consideration.

#### 3.1.1. Emotional Go/NoGo task performance

For IES, significant group differences between CD patients and controls were observed during both task trial conditions, with the CD group showing a lower speed-accuracy trade-off than their healthy counterparts (*p* < 0.001; Table 2). However, we did not find an association between RSA and IES (Table 3) in either task trial condition, and excluding participants taking medication with potential PNS effects did not alter these results (Table 4).

**Table 2:** Group differences in task performance and RSA.

	TD (*N* = 753)	CD (*N* = 693)	*t*-value	*p*-value
	Mean (SD)	Mean (SD)		
RSA	1.881 (0.237)	1.847 (0.246)	−0.645	0.519
IES Cognitive Regulation	0.049 (4.169)	−0.023 (3.511)	4.535	<0.001
IES Emotion Regulation	0.137 (3.279)	0.112 (3.531)	3.921	<0.001

Outcome of the regression models to investigate group differences between adolescent with CD diagnosis compared to TDs in the main variables of interest: RSA and the Inverse Efficiency Scores (IES) as a Speed-Accuracy trade off score of z-transformed mean reaction time (Go trials) and z-transformed correct response rate to NoGo trials (1-incorrect response rate to NoGo trials) in the emotion regulation and cognitive regulation conditions of the task. RSA results were computed using a multilevel model analysis including the following variable of interest: group, and the following variables of no interest which, based on our results in Supplementary Table S1 show an influence on RSA. These variables were age, medication intake and cigarettes smoked per day. IES results were computed using a *t*-test. Significance level = *p* < 0.05. Results for RSA show that when including variables shown to influence RSA, no significant group differences were found. For IES, significant group differences were found in both task trial conditions, emotion and cognitive regulation. During both task trial conditions, the TD group shows higher task performance than the CD group.

**Table 3:** Results of multi-level regression analyses on task performance measures.

	Value	Std. Error	DF	*t*-value	*p*-value
IES cognitive regulation					
RSA	0.083	0.104	1427.000	0.799	0.425
Group	−0.074	0.133	1427.000	−0.554	0.580
Age	0.093	0.112	1427.000	0.830	0.407
Interaction RSA × Group	0.018	0.102	1427.000	0.179	0.858
IES emotion regulation					
RSA	0.093	0.091	1427.000	1.020	0.308
Group	0.129	0.117	1427.000	1.105	0.269
Age	0.020	0.098	1427.000	0.200	0.842
Interaction RSA × Group	0.093	0.090	1427.000	1.037	0.300

The table shows the results of a multilevel regression analyses to investigate the association between RSA measures and performance measures in the Emotional Go/NoGo task. Key task performance measures were Inverse Efficiency Scores (IES) as a speed-accuracy trade off score of z-transformed mean reaction time (Go trials) and z-transformed correct response rate to NoGo trials (1-incorrect response rate to NoGo trials) in the emotion regulation and cognitive regulation conditions of the task. The multilevel models included additional fixed effects to control covariates for ADHD diagnosis, age, IQ, SES, sex, number of cigarettes smoked per day and as random effect site. All questionnaire scores were *t*-scored and centered and all variables included in the model were z-transformed. RSA, respiratory sinus arrhythmia measure at baseline; Group, difference between patient group CD and control group TD (reference group = TD); Std. Error, standard error; DF, degrees of freedom. Significance level = *p* < 0.05. No significant associations were found between RSA or RSA × Group interactions and task performance measures.

**Table 4:** Results of multi-level regression analyses on task performance measures after exclusion of participants with positive intake of medication with potential impact on PNS function.

	Value	Std.Error	DF	*t*-value	*p*-value
IES cognitive regulation					
RSA	0.070	0.115	1177.000	0.604	0.546
Group	−0.103	0.146	1177.000	−0.703	0.483
Age	0.094	0.120	1177.000	0.783	0.434
Interaction RSA × Group	0.004	0.115	1177.000	0.035	0.972
IES emotion regulation					
RSA	0.129	0.103	1177.000	1.251	0.211
Group	0.164	0.131	1177.000	1.256	0.210
Age	−0.034	0.108	1177.000	−0.312	0.755
Interaction RSA × Group	0.152	0.104	1177.000	1.470	0.142

The table shows the relationship between RSA and the different dependent variables in the study. Key task performance measures were Inverse Efficiency Scores (IES) as a speed-accuracy trade off score of z-transformed mean reaction time (Go trials) and z-transformed correct response rate to NoGo trials (1-incorrect response rate to NoGo trials) in the emotion regulation and cognitive regulation conditions of the task. Participants were excluded if a psychotropic medication is currently used. Medication intake was coded as a dichotomous variable (0 = no medication and 1 = medication intake). Models included additional fixed effects to control covariates for ADHD diagnosis, age, IQ, SES, sex, number of cigarettes smoked per day and as random effect site. All questionnaire scores were t-scored and centered, and all variables included in the model were z-transformed. RSA, respiratory sinus arrhythmia measure at baseline; Group, difference between patient group CD and control group TD (reference group = TD); Std. Error, standard error; DF, degrees of freedom. Significance level = *p* < 0.05. No significant associations were found between RSA or RSA × Group interactions and task performance measures.

To further investigate the role of internalising symptoms and CU traits in the association between RSA and emotion and cognitive regulation measures, we conducted post-hoc exploratory analyses including total subscale scores for internalising symptoms (CBCL) and total sum scores for CU traits (ICU) in the extended model (Table 5). We found no significant association between RSA and IES, and model comparison results suggested no significantly better model fit for the extended model (Table 6). Supplemental analysis comparing groups in correct response rates and reaction times revealed significant group differences (Supplementary Table S2). In the cognitive regulation condition, the TD group showed higher correct response rates and shorter reaction times than the CD group, and higher correct response rates and longer reaction times in the emotion regulation condition. Multilevel regression models in the MRI subsample (*N* = 577) did not find an association of RSA or group with IES (Supplementary Table S3).

**Table 5:** Results of multi-level regression analyses on task performance measures including internalising symptoms and CU traits in the model.

	Value	Std.Error	DF	*t*-value	*p*-value
IES cognitive regulation					
RSA	0.083	0.103	1425.000	0.804	0.422
Group	−0.232	0.159	1425.000	−1.458	0.145
Age	0.081	0.112	1425.000	0.722	0.471
Interaction RSA × Group	0.023	0.102	1425.000	0.225	0.822
IES emotion regulation					
RSA	0.092	0.091	1425.000	1.016	0.310
Group	0.084	0.139	1425.000	0.603	0.546
Age	0.015	0.098	1425.000	0.150	0.881
Interaction RSA × Group	0.094	0.090	1425.000	1.044	0.297

The table shows the relationship between RSA and the different dependent variables in the study including internalising symptoms and CU traits in the model. Key task performance measures were Inverse Efficiency Scores (IES) as a speed-accuracy trade off score of z-transformed mean reaction time (Go trials) and z-transformed correct response rate to NoGo trials (1-incorrect response rate to NoGo trials) in the emotion regulation and cognitive regulation conditions of the task. Models included additional fixed effects to control covariates for ADHD diagnosis, age, IQ, SES, sex, number of cigarettes smoked per day, internalising symptoms, CU traits and as a random effect site. RSA, respiratory sinus arrhythmia measure at baseline; Std. Error, standard error; DF, degrees of freedom; Internalising symptoms, total score of the internalising subscale of the parent reported version of the Child Behavior Checklist (CBCL); CU traits, total score of the parent reported version of the Inventory of Callous Unemotional traits (ICU). All questionnaire scores were *t*-scored and centered, and all variables included in the model were z-transformed. Significance level = *p* < 0.05. No significant associations were found between RSA or RSA × Group interactions and task performance measures.

**Table 6:** ANOVA model comparison: with and without including internalising symptoms and CU traits in the model.

	Model	df	AIC	BIC	logLik	Test	L.Ratio	*p*-value
IES cognitive regulation								
Simple model	1.000	12.000	8029.777	8093.095	−4002.888			
Extended model	2.000	14.000	8028.026	8101.898	−4000.013	1 vs. 2	5.751	0.056
IES emotion regulation								
Simple model	1.000	12.000	7659.742	7723.061	−3817.871			
Extended model	2.000	14.000	7662.819	7736.691	−3817.410	1 vs. 2	0.923	0.630

The table show the results of the ANOVA model comparison with and without including internalising symptoms and CU traits in the model. The dependent variables are the Inverse Efficiency Scores (IES) as a speed-accuracy trade off score of z-transformed mean reaction time (Go trials) and z-transformed correct response rate to NoGo trials (1-incorrect response rate to NoGo trials) in the emotion regulation and cognitive regulation conditions of the task. The simple model included fixed effects of RSA, ADHD diagnosis, age, IQ, SES, sex and number of cigarettes smoked per day. The extended model included the same fixed effects as in the simple model with additional fixed effects of internalising symptoms and CU traits. The random effect in all models was the data collection site. Model, number of models included for the model comparison; df, degrees of freedom; AIC, Akaike information criterion; BIC, Bayesian information criterion; logLik, log-likelihood; Test, indicating which models are being compared; L.Ratio, Likelihood-ratio test; internalising symptoms, total score of the internalising subscale of the parent reported version of the Child Behaviour Checklist (CBCL); CU traits, total score of the parent reported version of the Inventory of Callous Unemotional traits (ICU). All questionnaire scores were *t*-scored and centered, and all variables were z-transformed. Significance level = *p* < 0.05. For IES in both task trial conditions, the extended model did not significantly improve fit to the data.

### 3.2. Structural imaging results

**Figure 1:**
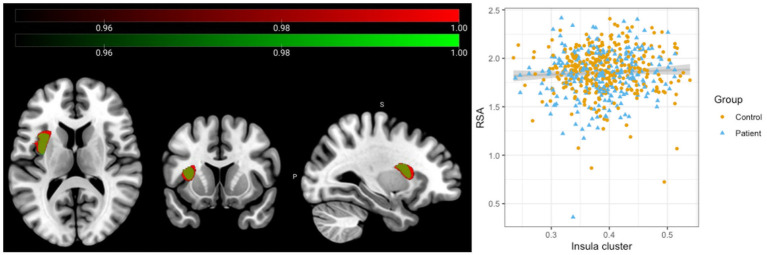
Association between Respiratory Sinus Arrhythmia and Insular grey matter volume across all participants. Results of the full factorial analyses with standardized RSA values included as the main regressor of interest and total intracranial volume (TIV), age, IQ and site as regressors of no interest. A significant positive association was observed across all participants between grey matter volume in the left insula and RSA values (Red cluster, *N* = 577, MNI coordinates: −34; 4; 13; 3,417 mm^3^, pFWE = 0.011). Repeating the analysis only including those participants with higher image quality did not change the results significantly (Green cluster, *N* = 462, MNI coordinates: −34, 2, 15; 2,597 mm^3^, pFWE = 0.034).

Full factorial analyses showed a significant association between grey matter volume in the left insula and RSA values across all participants (Figure 1, red cluster, pFWE = 0.011, MNI coordinates: −34; 4; 13, 3,416 mm^3^). There were no significant group, sex or RSA × group interaction effects. To test whether these results would remain significant when only those individuals with higher image quality were included, the analysis was repeated with those participants with image quality B or higher. Results remained essentially unchanged (Figure 1, green cluster, pFWE = 0.034, MNI coordinates: −34, 2, 15; 2,597 mm^3^).

## 4. Discussion

The current study investigated the association between RSA (as indicator of heart rate variability) and neuropsychological measures of emotion and cognitive regulation in a sample of adolescents with and without a diagnosis of CD. Our results show that RSA was positively associated with grey matter volume in the left insular cortex across all participants, but there were no significant differences between healthy adolescents and those with diagnosis of CD in the strength of the association. However, RSA was not associated with task performance in either cognitive or emotion regulation trials.

Across all participants, individual differences in the specified measure for HRV, RSA, were positively associated with the left insula, key region for emotion and cognitive regulation. Activation in the anterior insula has been associated with sympathetic and parasympathetic activation across a variety of tasks including cognitive, affective and somato-sensory tasks (77), linked to increased autonomic arousal during task performance, and suggested as a potential major site for visceral representations (78). Our results are thus also in line with previous suggestions linking structural and functional abnormalities in the insula with psychopathology (79–81) also showing deficits in the affective and cognitive dimensions of executive function (82).

While the available evidence seems to suggest that right-lateralized neural inputs might be more relevant than their contra-lateral homologous regions for HRV regulation (32, 34, 77) our results show an association across all participants with the left anterior insular cortex volume. This brain region has been implicated in both emotion (83) and cognitive regulation processes (84). As part of the salience network, the insula mediates interactions between other large-scale networks such as the default mode network and central executive network (85). Thus, difficulties in switching between neural circuits in response to environmental demands, also linked with low HRV (14, 86) may indicate a vulnerability to generic self-regulation deficits. However, no significant differences between groups were observed. Thus, the observed association between brain structure and HRV might constitute a vulnerability factor for difficulties in self-regulation, contributing to their subsequent manifestation.

Previous studies have found significant negative associations between insular volumes or thickness and HRV measures in healthy adults (34). In addition, a recent meta-analyses has shown a significant positive association between cortical thickness and HRV measures in a number of regions including lateral orbitofrontal cortex and insular cortex, declining with age (87). However, in this study data of adolescents and young adults (18 year-olds) were analysed together under the assumption of linear association and therefore any potential quadratic trajectories in this association (potentially showing an inverted-U shape) might have been missed. We however, observed a significant positive association, potentially related to neurodevelopmental processes that are still undergoing during adolescence (88, 89). Given the role of the ANS system in supporting the development of the prefrontal cortex (55), further studies will be needed to elucidate the longitudinal differences with increasing age. The protracted maturational processes of crucial prefrontal regions for emotion and cognitive regulation including insular cortex increase the likelihood of difficulties that might contribute to the observed psychopathology (90). The insular body has indeed shown a quadratic developmental trajectory, with increasing cortical thickness during the first two decades of life and decreasing thereafter (89), a trajectory that mirrors the one described for HRV measures (55, 91). On older individuals, associations with cortical thickness on lateral OFC and ACC might be more evident (32, 87, 92). Other studies on the other hand have found no associations between HRV and cortical volumes in healthy adult samples but links with functional connectivity instead (35). This might be relevant given the prominent connectivity patterns described between the anterior and middle insula regions and dorsal anterior cingulate cortex (93).

While our results suggest no significant association between HRV and different behavioural measures of self-regulation, it might potentially be subject to several individually varying factors such as sex or age or to differences in the sensitivity of different HRV measures (52). In addition, although RSA is commonly used to quantify HRV, there is some debate about its sensitivity, whether correction for heart rate or respiration is necessary (94), with other measures of HRV as potential alternatives (36). Furthermore, there is some evidence from adolescents with self-injury behaviours where no association between baseline or reactivity of HRV measures was shown, but only on recovery processes, indicative of a poor ability to regulate response to stressors (28), or an association between HRV recovery but not at baseline with specific cognitive functions (95), a lack of association between inhibitory scores and basal HRV measures but with reactivity during inhibitory performance in preschool-aged children with early adverse experiences (96), or differentiated associations as a function of psychopathological profiles (97). One significant limitation is that our psychophysiological measures of HRV were only acquired at rest. Future studies should ideally combine measures of cardiovagal function at rest with the investigation of phasic changes in HRV within the same individuals, including both reactivity (response during stressors or challenging situations) and recovery (function after stressors) capacities (1), as well as potentially changes in reactivity over time (98). This would possibly provide a more complete picture of the association between HRV and self-regulation behaviours.

According to de Looff et al. (22), psychophysiological effects are also dependent on the experimental task, parameters, and analyses. In addition, while the Emotional Go/NoGo task has been shown to measure both emotion and cognitive regulation (66, 99), the “baseline” condition for the cognitive control condition are emotional facial expressions, therefore requiring the processing of facial emotions. The lack of differences between CD youth and healthy controls in the Emotional Go/NoGo task might be due to the inclusion of emotional faces in both emotional and cognitive regulation task conditions, which might interfere with the elicitation of these regulatory processes distinctively enough. To further investigate the specificity of the association between HRV and emotion regulation, future studies using cognitive control tasks not involving facial emotion processing would be needed.

The results of the extended model after inclusion of internalising problems and CU traits scores suggest that the clinical and symptomatologic heterogeneity of the group of CD participants might have significantly contributed to the difficulty to identify potential group differences in the task. This is in line with previous studies suggesting psychophysiological heterogeneity within patients with antisocial and aggressive behaviours (22, 31). Thus, potential differences between patients and their healthy counterparts might be easier to identify when such symptomatic differentiations within patients is taken into consideration.

In conclusion, we found a positive association between RSA and gray matter volumes in the left anterior insula. This region has been shown to be involved in emotion and cognitive regulation processes, suggesting that HRV is not solely linked with emotion regulation capacities but more with generic self-regulation processes. Since structural and functional abnormalities of the insula have been linked to many mental disorders including CD, the observed association between brain structure and HRV might constitute one risk factor that, in combination with others, might lead to self-regulation difficulties. As the insula also mediates the switching among different neural circuits in response to environmental demands (84) these processes might be affected in the case of low RSA and associated smaller grey matter volumes. Thus, further research should focus on network dysfunctions rather than individual brain regions, the additional use of HRV reactivity and recovery measures in combination with other ANS indices and the use of paradigms measuring clearly differentiated self-regulation aspects. This might then contribute to provide a clearer picture of the neural mechanisms underlying the association between individual differences in HRV and self-regulation deficits.

## Data availability statement

The datasets presented in this article are not readily available because the datasets are from the FemNAT-CD multi-country consortium and are available on reasonable request in line with the consortiums’ data sharing policy. Requests to access the datasets should be directed to CS (christina.stadler@upk.ch).

## Ethics statement

The studies involving human participants were reviewed and approved by the ethics committee of the medical faculty of the Goethe- University in Frankfurt am Main, Germany. After the coordinating site of the multicenter study, Goethe University Frankfurt, received the approval, local ethics committees from all other sites approved, too. Written informed consent to participate in this study was provided by the participants’ legal guardian/next of kin.

## Author contributions

AT and AC designed the project and performed the analysis. HO and EU verified the analytical methods. CS supervised the project. HO, NR, GK, LN-J, AH, AF-R, KK, AP, CF, SB, and GF collected and provided the data. AT and AC wrote the paper with input from all authors. All authors contributed to the article and approved the submitted version.

## Funding

This study was supported by the European Commission under the 7th Framework Programme [FemNAT-CD, coordinator CF; grant agreement number 602407]. AT was supported by the Swiss National Science Foundation (Grant no. 100014_185408/1).

## Conflict of interest

The authors declare that the research was conducted in the absence of any commercial or financial relationships that could be construed as a potential conflict of interest.

## Publisher’s note

All claims expressed in this article are solely those of the authors and do not necessarily represent those of their affiliated organizations, or those of the publisher, the editors and the reviewers. Any product that may be evaluated in this article, or claim that may be made by its manufacturer, is not guaranteed or endorsed by the publisher.

## Footnotes

^1^ https://www.femnat-cd.eu
