# Supplementary material for: Linking heart rate variability to psychological health and brain structure in adolescents with and without conduct disorder
Source: Front Psychiatry. 2023 Jun 27;14:1101064. doi: 10.3389/fpsyt.2023.1101064 (PMC10333527; doi:10.3389/fpsyt.2023.1101064)
Supplement: Supplementary file 1 [file Table_1.docx]

***Supplementary Material***

# Supplementary Tables

**Supplementary Table S1. Results of multi-level regression analyses on RSA**

|  |  |  |  |  |  |
| --- | --- | --- | --- | --- | --- |
|  | *Estimate* | *Std. Error* | *df* | *t-value* | *p-value* |
| Age | -0.132 | 0.029 | 1429.000 | -4.516 | < 0.001 |
| Sex | 0.005 | 0.028 | 1429.000 | 0.171 | 0.864 |
| BMI | 0.023 | 0.028 | 1429.000 | 0.830 | 0.407 |
| SES | -0.029 | 0.027 | 1429.000 | -1.073 | 0.284 |
| Cigarettes/day | -0.056 | 0.028 | 1429.000 | -2.006 | 0.045 |
| Sports (h)/week | 0.034 | 0.027 | 1429.000 | 1.267 | 0.206 |
| PNS Medication intake | -0.081 | 0.027 | 1429.000 | -3.022 | 0.003 |

This table shows the results of the multilevel regression analysis including variables possibly influencing RSA based on previous findings in the literature (e.g. Prätzlich et al., 2019; Oldenhof et al., 2019). The following independent variables were included in the model as fixed effects: Age, sex, BMI (Body-Mass-Index), SES = Socio economic status based on parental income, education and occupation, cigarettes smoked per day, sports hours per week, medication intake and as random effect: site. Std. Error = Standard Error, df = degrees of freedom. Significance level = p < 0.05. Results showed significant negative effects of age, cigarettes and medication intake on RSA.

**Supplementary Table S2. Group differences in Correct Responses and Reaction Time**

|  | **TD (N=753)** | **CD (N=693)** | *t-value* | *p-value* |
| --- | --- | --- | --- | --- |
|  | *Mean (SD)* | *Mean (SD)* |  |  |
| Correct response rate Cognitive Regulation | 68.746 (1.946) | 64.463 (2.066) | -13.245 | < 0.001 |
| Reaction time Cognitive Regulation | 422.499 (62.283) | 423.134 (71.282) | -240.68 | < 0.001 |
| Correct response rate Emotion Regulation | 66.585 (1.844) | 60.256 (2.048) | -11.063 | < 0.001 |
| Reaction time Emotion Regulation | 428.626 (72.073) | 424.774 (67.89) | -231.24 | < 0.001 |

This table shows group differences between adolescents with CD diagnosis compared to TDs in RSA and performance measures in the Go/NoGo task which are reaction times to Go trials and proportion of correct response rates to NoGo trials (1- incorrect response rate to NoGo trials) during the emotion regulation and cognitive regulation condition of the Emotional Go/NoGo task. Correct response rate to NoGo trials (%), Reaction time (ms). Significance level = p < 0.05. The TD group shows both higher correct response rates and shorter reaction times than the CD group during cognitive regulation trials. During emotion regulation trials the TD group shows higher correct response rates and longer reaction times than the CD group.

**Supplementary Table S3: Results of multi-level regression analyses on task performance measures in the subsample with T1 imaging data (N=577)**

| IES Cognitive Control | | | | | |
| --- | --- | --- | --- | --- | --- |
|  | *Value* | *Std.Error* | *DF* | *t-value* | *p-value* |
| RSA | -0.101 | 0.160 | 563.000 | -0.628 | 0.530 |
| Group | -0.391 | 0.209 | 563.000 | -1.866 | 0.063 |
| Age | -0.011 | 0.170 | 563.000 | -0.065 | 0.948 |
| Interaction RSAxGroup | -0.112 | 0.160 | 563.000 | -0.700 | 0.484 |
| IES Emotion Control | | | | | |
|  | *Value* | *Std.Error* | *DF* | *t-value* | *p-value* |
| RSA | -9.217 | 17.409 | 562.000 | -0.529 | 0.597 |
| Group | -24.600 | 22.622 | 562.000 | -1.087 | 0.277 |
| Age | -139.454 | 18.479 | 562.000 | -7.546 | 0.000 |
| Interaction RSAxGroup | -14.579 | 17.424 | 562.000 | -0.837 | 0.403 |

This table shows the relationship between RSA and the different dependent variables in the study in the subsample of participants included in the imaging analysis. Key task performance measures were Inverse Efficiency Scores (IES) as speed-accuracy trade off scores of z-transformed mean reaction time (Go trials) and z-transformed correct response rate to NoGo trials (1-incorrect response rate to NoGo trials) in the emotion regulation and cognitive regulation conditions of the task. Models included additional fixed effects to control covariates for ADHD diagnosis, age, IQ, SES, sex, number of cigarettes smoked per day and as random effect site. All questionnaire scores were t-scored and centered, and all variables included in the model were z-transformed. RSA = Respiratory Sinus Arrhythmia measure at baseline, Group = difference between patient group CD and control group TD (reference group = TD), Std. Error = Standard Error, df = degrees of freedom. Significance level = p < 0.05. No significant associations were found between RSA or RSA x Group interactions on task performance measures.

## Supplementary Figures

**Supplementary Figure S1. Masked regions of the CAN (Central Autonomic Network)**

For a Region Of Interest (ROI) analysis we created a mask consisting of regions of the CAN which regulate ANS activity but are also involved in emotional and cognitive self-regulation processes (i.e., amygdala, insular cortex, anterior cingulate cortex & ventromedial prefrontal cortex).
