# Supplementary figures and images for: Linking heart rate variability to psychological health and brain structure in adolescents with and without conduct disorder
Source: Front Psychiatry. 2023 Jun 27;14:1101064. doi: 10.3389/fpsyt.2023.1101064 (PMC10333527; doi:10.3389/fpsyt.2023.1101064)

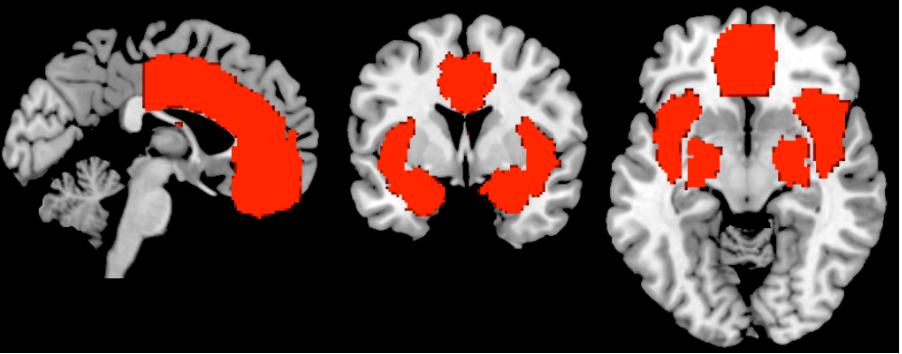

Supplement: Supplementary file 2 [file Image_1.TIFF]
